# The Habits of the Medical Student Past and Present

**Published:** 1907-10-05

**Authors:** 


### Resident Medical Officers' Department.

[Contributions to this Column are invited, and, if accepted, will be paid for.]

### THE HABITS OF THE MEDICAL STUDENT PAST AND PRESENT.

Thirty, twenty, even fifteen years ago the popular conception of a medical student was that of a riotous, gregarious, impecunious, and carelessly garbed youth who did little work and spent most of his evenings smoking, drinking, and being ejected from music halls and various Alsatian resorts; who played practical jokes and did his best to paint the town red, as it was called. No doubt this idea was based to a greater or less extent upon facts; at any rate it was so deeply rooted that most young men, from University "bloods" to linendrapers' assistants, when charged by the police with any kind of alcoholic exuberance, cheerfully described themselves as medical students. And this occupation was held to explain quite naturally, if not actually to condone, those particular forms of misconduct.

To some people the passing away of this order has seemed a thing to be regretted, a symptom of degeneration from healthy vitality into premature priggishness. Of the passing itself there is no reasonable doubt. The medical student of to-day is everywhere, and in London especially, unrecognisable as the original of the portrait we have drawn, or of those left us by the satirists of the mid-Victorian period.

The reasons are not very difficult to find. The whole tendency of medical education is towards greater complexity and efficiency. The standard of the preliminary arts examination is higher; therefore the age at which medical study is commenced is a later one. Indeed, it now averages 19 years 3 months, and numerous idlers are thus excluded from the very start. The curricula are longer and more exacting; therefore the average period of study is longer and the average student is older than in former times. At the same time the examinations are so much more difficult that increased application is necessary to face them successfully. The minimum period of medical study, which is now five years instead of four as was the case until some 15 years ago, is seldom long enough even for a hard-working student. The mean period in England is seven years five months, and rather less in Scotland and Ireland. That figure is drawn, of course, from the records of those who pass; when it is recalled that not more than half of those who register as students ever succeed in registering as practitioners, it is plain enough that the future medical man has nowadays too much to do to get into any serious mischief, or at least that he has not the idle hands that are supposed to conduce to it.

The modern student realises that if he is to obtain a qualification before middle age he must devote himself to his work with an energy that leaves little surplus for nocturnal encounters with the police. Thirty years ago a moderately intelligent young man could afford such escapades and their consequences without damaging his chances of passing his examinations in a reasonable time. In those days he began medical study younger, spent a shorter time over it, and worked less strenuously during it. He had more time and more temptation to be boyish, and he was in years much more a boy.

Another factor, in London at any rate, is the growth of the medical schools of the older universities: many men now come thence to London for the strictly professional part only of their studies. They are often somewhat satiate of mere amusement and anxious to qualify, and they are, of course, older than the general average of those who study in London schools from the beginning. In due time they occupy a large number of the house officerships at the teaching hospitals, and their influence and example is bound to affect for good the standard of conduct among the younger men.

We do not share the view that increased sobriety and decorum among medical students necessarily means priggery or snobbishness, or that increased professional knowledge and efficiency need make the medical man less fit for the general duties of citizenship. On the contrary, we regard the change in the character of the medical student as most important and most necessary for that dignity of the profession in the future, and that standing amongst the community which, before it will be conceded, must be earned by high standards of conduct and ability. The various forms of athletic exercise are not difficult to pursue in England; some of the best, for instance rowing, are even in some danger of neglect; and the recreation and relaxation which the medical student needs, and ought to have, are better obtained in healthy sport than in some other ways which have steadily and deservedly lost the favour of the class to which they were once supposed particularly to appeal.

